# Investigating the Contribution of Molecular-Enriched Functional Connectivity to Brain-Age Analysis

**DOI:** 10.1101/2025.10.16.682939

**Published:** 2025-10-16

**Authors:** Marco Pinamonti, Manuela Moretto, Valentina Sammassimo, Marco Castellaro, Mattia Veronese

**Affiliations:** 1 Department of Information Engineering, University of Padua, via Gradenigo 6/b, Padua, 35131, Italy; 2 Institute of Psychiatry, Psychology & Neuroscience, Department of Neuroimaging, King’s College London, 16 De Crespigny Park, London SE5 8AB, United Kingdom

**Keywords:** brain-age, molecular-enriched functional connectivity, MRI

## Abstract

Brain-age prediction from neuroimaging data provides a proxy of biological aging, yet most models rely on structural magnetic resonance imaging (MRI), a modality that captures macroanatomy but offers limited biological specificity. We tested whether integrating molecular-enriched functional connectivity (FC), from resting-state functional MRI (rs-fMRI) data, improves brain-age prediction and biological explainability.

We analyzed MRI data of 2,120 healthy adults (1,243/877 F/M; 18–90 years) from three public datasets. Molecular-enriched connectivity maps were derived with Receptor-Enriched Analysis of functional Connectivity by Targets (REACT) using receptor-density templates for the dopamine (DAT), norepinephrine (NET), and serotonin (SERT) transporter systems. Support vector regression models were applied to predict chronological age from molecular-enriched FC, structural morphometry, or both combined. The effect of multi-site variability was mitigated via ComBat harmonization with and without Empirical Bayes pooling. We additionally conducted a common-parcellation analysis to assess the impact of differing parcellations between modalities.

Single-transporter molecular-enriched FC explained up to 51% of age variance. The most predictive transporter varied by dataset, with DAT dominating in the harmonized and common-parcellation settings. Combining the three molecular-enriched maps consistently improved prediction over any single map and increased explained variance up to 64%. In the merged multi-site cohort using a common parcellation, augmenting structural information with transporter-enriched FC reduced mean absolute error (MAE) from 6.02 to 5.81 years, supporting complementarity of the two modalities. In contrast, when different parcellations were applied, incorporating molecular-enriched FC into brain age prediction resulted in a 2% higher MAE compared to structural morphometry alone, suggesting that parcellation mismatch may obscure the functional contributions.

In conclusion, molecular-enriched FC is a feasible and biologically informative extension to brain-age modeling, enhancing prediction and interpretability with respect to neurotransmitter systems.

## 1 Introduction

Worldwide, life expectancy has risen dramatically over recent decades; demographic forecasts indicate that by 2050, approximately 16% of the global population will be older than 65 years, nearly double the 9% recorded in 2019 (United Nations, Department of Economic and Social Affairs, Population Division, 2019). This demographic shift is already imposing major challenges for sustainability of healthcare systems and social services, as older age is accompanied by a higher burden of chronic diseases and disabilities (World Health Organization, 2024). The clinical and public health implications are urgent and far-reaching, reflecting a rapidly growing global burden and escalating economic costs that motivate sensitive markers of aging and early risk stratification (Alzheimer’s Disease International, 2024; Livingston et al., 2024).

Aging is a complex physiological process characterized by the gradual accumulation of molecular and cellular damage, leading to functional decline and increased vulnerability to disease (Blinkouskaya et al., 2021). Advanced age represents the greatest risk factor for numerous chronic conditions, including cancers, cardiovascular disease, and neurodegenerative disorders such as dementia and Alzheimer’s disease (MacDonald & Pike, 2021). Yet the rate at which aging unfolds varies markedly among individuals, reflecting a dynamic interplay of genetic, lifestyle, and environmental influences (Moskalev, 2019; Udeh-Momoh et al., 2025). Consequently, chronological age often fails to capture an individual’s biological state. The concept of biological age has therefore gained traction as a more informative metric across organ systems, with molecular and phenotypic clocks used to stratify risk and to monitor intervention effects (Moskalev, 2019).

Within this framework, neuroimaging has been widely used to estimate the biological age of the brain, which is thought to reflect brain-specific aging processes (Cole & Franke, 2017; Franke & Gaser, 2019). Often referred to as “brain-age”, this framework consists of training machine-learning models on large normative magnetic resonance imaging (MRI) datasets of T1-weighted structural images to predict chronological age (Cole et al., 2015; Franke et al., 2010). Hence for a new individual, the brain-age gap (sometimes also referred to as the brain-age Δ) can be calculated as the difference between the predicted brain-age and the person’s actual chronological age. A positive Δ indicates an older-appearing brain than expected for chronological age, whereas a negative Δ indicates a younger-appearing brain. In applications, Δ can be summarized for group comparisons (e.g., patients vs. controls) or tracked longitudinally within individuals for risk stratification and to gauge intervention effects. This metric is typically interpreted as an index of accelerated or decelerated brain aging: large positive Δ is associated with diverse disorders, including schizophrenia (Koutsouleris et al., 2013; Nenadić et al., 2017; Schnack et al., 2016), dementia (Wang et al., 2019), Alzheimer’s disease (Franke & Gaser, 2012), multiple sclerosis (Høgestøl et al., 2019), epilepsy (Pardoe et al., 2017), HIV infection (Cole, Underwood, et al., 2017), Down syndrome (Cole, Annus, et al., 2017), and type 2 diabetes (Franke et al., 2013). On the contrary, healthy habits in terms of diet and environment have been shown to slow the aging process in animal models, resulting in negative Δ (Brusini et al., 2022).

To enhance the sensitivity and accuracy of brain-age models, functional connectivity (FC) features derived from resting-state functional MRI (rs-fMRI) have been incorporated (Liem et al., 2017). This follows evidence from numerous studies showing that normal aging is characterized by systematic alterations in network coherence and inter-network communication, assessed using both static (Betzel et al., 2014; Ferreira & Busatto, 2013) and dynamic approaches (Chen et al., 2019; Moretto et al., 2022; Tian et al., 2018; Xia et al., 2019). On the basis of these age-related alterations in resting-state FC (rs-FC) features, a few studies have attempted to predict chronological age using machine-learning models trained on rs-FC patterns (Bi et al., 2024; Millar et al., 2022). In Bi et al. (2024), age was predicted with a mean absolute error (MAE) of 6–7 years, with predictions explaining approximately 74% of age variability, approaching but not surpassing the precision of structural MRI–based models that stand at 93% (Cole et al., 2019; Gutiérrez Becker et al., 2018). Notably, in preclinical Alzheimer’s disease, deviations in FC-based brain age have been detected even before overt memory symptoms emerge (Millar et al., 2022), suggesting that rs-FC may capture early functional changes missed by structural imaging.

The BOLD signal reflects changes in the local balance of oxy- and deoxyhemoglobin arising from neurovascular coupling and thus provides an indirect hemodynamic proxy for neural activity. Consequently, despite its potential for studying brain age, rs-fMRI lacks neurochemical specificity (Logothetis, 2008). To address this limitation, Dipasquale et al. (2019) introduced the Receptor-Enriched Analysis of functional Connectivity by Targets (REACT), which integrates BOLD fluctuations with normative receptor-density maps derived from PET or SPECT, yielding molecular-enriched functional connectivity that incorporates neurochemical information. After its introduction, REACT has been increasingly applied in diverse contexts, including pharmacological challenges such as MDMA (Dipasquale et al., 2019; Singleton et al., 2023), LSD (Lawn et al., 2022), and methylphenidate (Dipasquale et al., 2020); neurological conditions such as multiple sclerosis (Cercignani et al., 2021), Parkinson’s disease (Di Vico & Moretto et al., 2025), and visual snow syndrome and migraine with aura (Puledda et al., 2023); as well as in chronic pain (Martins et al., 2021). Moreover, by integrating REACT with normative modeling across the healthy lifespan, Lawn et al. (2024) showed that molecular-enriched network deviations systematically track age-related functional reorganization, providing a sensitive molecularly informed biomarker of brain-age (Lawn et al., 2024). Integrating such molecularly informed functional features with established structural metrics therefore holds promise for refining brain-age estimates and elucidating the neurochemical substrates of age-related brain changes.

Building molecular-enriched rs-fMRI into brain-age paradigms comes at the cost of a few methodological challenges. First and foremost, cohorts must be large enough (N>2,000) to match the sample sizes that have made structure-only models successful (Han et al., 2020; Varoquaux, 2018; Yu et al., 2024). Because single datasets rarely reach this scale, assembling an adequately powered cohort requires aggregating data across sites, scanners, and protocols. Such aggregation demands principled harmonization to attenuate non-biological site effects while preserving age-related signal. In parallel, study design should support generalizability by employing broad normative training samples, enforcing consistent parcellation across modalities, and evaluating performance across sites and acquisition conditions.

Motivated by the need for biologically informative features, the present study investigates whether applying REACT to integrate neurochemical information into rs-fMRI data from 2,120 healthy adults might improve brain-age prediction. Specifically, it aims to (i) quantify the standalone prediction accuracy of molecular-enriched FC and benchmark it against structural T1-weighted (T1w) MRI — evaluating each modality, both separately and combined, under the hypothesis that molecular-enriched FC provides complementary variance that enhances overall accuracy — and (ii) assess the multi-site problem by comparing alternative harmonization strategies for mitigating scanner and site effects across structural morphometry, molecular-enriched FC, and receptor-density atlas features within a unified modeling pipeline.

## 2 Methods

### 2.1 Ethics statement

This study analyzed de-identified human neuroimaging and phenotypic data from three publicly available adult cohorts. Each source study obtained approval from its local research ethics committee/institutional review board, and all participants provided written informed consent for data collection and for data sharing. Data access and use complied with each resource’s data-use terms; no attempts at re-identification were made, and all reporting is at the group level. No new data were collected, and no animal research was involved. Receptor-density templates used in REACT were taken from previously published PET/SPECT atlases that were acquired under the oversight of their originating studies’ ethics committees and reused under their licenses. All procedures adhered to the Declaration of Helsinki and applicable data-protection regulations.

### 2.2 Datasets

This study assembled a large cohort of normative individuals with both rs-fMRI and T1w MRI by pooling three publicly available lifespan studies focused on aging: the “Cambridge Centre for Ageing and Neuroscience” study (Cam-CAN) (Shafto et al., 2014; Taylor et al., 2017), the “Lifespan Human Connectome Project in Aging” (HCP-Aging) (Bookheimer et al., 2019; Harms et al., 2018), and the “Nathan Kline Institute Rockland Sample” (NKI-RS) (Nooner et al., 2012).

Across these cohorts, the “healthy” status of participants was defined pragmatically for normative aging research as community-dwelling adults who could provide informed consent and safely undergo MRI. Exclusions focused on major diagnosed disease across neurological (e.g., stroke, Parkinson’s disease, epilepsy), psychiatric (e.g., schizophrenia, bipolar disorder), and systemic medical domains (e.g., active cancer therapy, uncontrolled cardiovascular disease), as well as cognitive impairment, current substance misuse, and MRI contraindications; common, stable age-related conditions (e.g., well-controlled hypertension) were generally permitted. Cam-CAN and HCP-Aging explicitly screened for major neurological and psychiatric illness within this “typical aging” framework, whereas NKI-RS used community-ascertained recruitment calibrated to the age, sex, ethnicity, and socioeconomic profile of Rockland County, New York, to approximate broader U.S. demographics (Bookheimer et al., 2019; Nooner et al., 2012; Shafto et al., 2014).

From these datasets, we included only participants with good quality T1w and rs-fMRI data resulting in 653 individuals from Cam-CAN, 724 from HCP-Aging and 978 from NKI-RS. For all these studies, imaging was acquired on 3 T Siemens systems with 32-channel head coils (TIM Trio for Cam-CAN and NKI-RS, Prisma for HCP-Aging). The multi-site study (HCP-Aging) implemented harmonized, site-matched sequences across four centers; Cam-CAN and NKI-RS were collected at single sites using consistent protocols. Sequence parameters are summarized in Table 1 (structural) and Table 2 (functional).

### 2.3 Image analysis

Across cohorts, we obtained raw T1w and rs-fMRI images and applied a uniform preprocessing workflow to ensure cross-cohort comparability. Raw images were organized according to Brain Imaging Data Structure (BIDS) specifications (Gorgolewski et al., 2016) and then processed with standardized BIDS Apps (BIDS Apps Community, 2025). Each dataset was then checked with the bids-validator (v1.14.6)^1^, which confirmed full compliance with the specification (Blair et al., 2024).

#### 2.3.1 Quality control of raw images

The large number of scans downloaded from Cam-CAN, HCP-Aging, and NKI-RS precluded exhaustive manual inspection; therefore, image quality was assessed with MRIQC^2^ (v24.1.0) (Esteban et al., 2017). MRIQC has become a standard tool for automated QC of structural and functional MRI data (Botvinik-Nezer et al., 2019; Nakua et al., 2023; Nitsch et al., 2024; Provins et al., 2023). The tool was run independently for each cohort and modality, and the resulting group-level reports were inspected. Any scan with image-quality metrics (IQMs) outside the inner Tukey fences (i.e., below *Q*1−1.5,IQR or above *Q*3+1.5,IQR) was flagged for review according to the non-parametric outlier rule (Tukey, 1977). We focused on IQMs that are particularly sensitive to motion and other artifacts: for T1w images, signal-to-noise ratio (SNR), gray–white matter contrast-to-noise ratio, and the entropy-focus criterion; for rs-fMRI runs, temporal SNR, mean framewise displacement, and temporal derivative of the variance of the BOLD signal (DVARS). All flagged scans were visually inspected; scans exhibiting motion, ringing, cropping or other obvious defects were excluded, and the remaining data were processed further.

#### 2.3.2 Preprocessing

T1w and rs-fMRI images were processed with a three-stage workflow that combined fMRIPrep (v23.2.2), XCP-D (v0.7.4), and FSL (v7.3.2). fMRIPrep is an automated, BIDS-compatible pipeline that delivers analysis-ready structural and functional derivatives by integrating core routines from Advanced Normalization Tools (ANTs), FMRIB Software Library (FSL), FreeSurfer (FS), and Analysis of Functional NeuroImages (AFNI) (Esteban et al., 2019, 2020); XCP-D is a post-processing toolkit that implements state-of-the-art nuisance regression and connectivity derivatives (Mehta et al., 2024); and FSL is a longstanding MRI toolbox offering spatial filtering, registration, and statistical modeling (Jenkinson et al., 2012; Smith et al., 2004). Together, these tools constitute one of the most widely adopted preprocessing stacks in contemporary neuroimaging studies (Bowring et al., 2019; Dugré et al., 2025).

##### Structural and functional preprocessing with fMRIPrep.

Each participant’s T1w image was corrected for intensity non-uniformity with N4 bias field correction (ANTs 2.5.0) (Tustison et al., 2010), then skull-stripped via the Nipype implementation of antsBrainExtraction.sh using the OASIS30ANTs target (TemplateFlow v23.1.0) (Ciric et al., 2022). Tissue segmentation into cerebrospinal fluid (CSF), white matter (WM), and gray matter (GM) was performed on the brain-extracted T1w with fast (FSL 7.3.2) (Zhang et al., 2001). Cortical surfaces were reconstructed with recon-all (FreeSurfer 7.3.2) (Fischl, 2012), and the initial brain mask was refined by reconciling ANTs- and FreeSurfer-derived GM segmentations via a Mindboggle-based algorithm (Klein & Tourville, 2012). Finally, the T1w was nonlinearly normalized to the MNI ICBM 152 NL-6 Asym template (2 mm isotropic) using antsRegistration (ANTs 2.5.0) through TemplateFlow (Fonov et al., 2011).

For each rs-fMRI run, a reference volume was generated for head-motion correction, and motion parameters were estimated with mcflirt (FSL) before any filtering (Jenkinson et al., 2012). The BOLD reference was coregistered to the T1w via boundary-based registration (bbregister, FreeSurfer) (Greve & Fischl, 2009), with six degrees of freedom. All spatial transformations (head-motion, BOLD to T1w, T1w to MNI) were composed and applied in a single interpolation step using nitransforms with cubic B-spline interpolation.

From fMRIPrep we retained only: (i) spatial transforms and the preprocessed BOLD in native and MNI spaces, and (ii) the _confounds.tsv columns needed to identify motion-affected frames (framewise displacement (FD) and DVARS) plus six rigid-body parameters (with their temporal derivatives and quadratics). Volumes with FD>0.5 mm or DVARS>1.5 *σ* were flagged for interpolation downstream (Power et al., 2012); no temporal filtering or nuisance regression was performed at this stage.

##### Post-processing with XCP-D.

The eXtensible Connectivity Pipeline for DCAN (XCP-D) was used to post-process fMRIPrep derivatives (Gorgolewski et al., 2011; Mehta et al., 2024). Confound regression followed the “acompcor” nuisance model, defined as six motion parameters plus their temporal derivatives and the top five aCompCor components from WM and CSF (Behzadi et al., 2007; Satterthwaite et al., 2013). Frames flagged as high-motion were cubic-spline interpolated; both data and confounds were then high-pass filtered (second-order Butterworth, 0.001 Hz cutoff) and denoised via linear regression of the nuisance set and cosine bases (Ciric et al., 2018). Because downstream analyses required continuous time series, we retained the interpolated, non-censored residual BOLD image produced by XCP-D (no spatial smoothing in this step).

##### Spatial smoothing with FSL.

The interpolated residual BOLD images were spatially smoothed in FSL 7.3.2 with a Gaussian kernel with an FWHM of 6 mm. These smoothed, denoised BOLD volumes were used for all subsequent FC and brain-age analyses.

### 2.4 Molecular-enriched functional connectivity

Using the preprocessed BOLD time series as input, we derived molecular-enriched FC maps that fuse neurotransmitter-specific information from positron-emission tomography (PET)/single-photon emission computed tomography (SPECT) templates with rs-fMRI, thus augmenting the hemodynamic proxy of neuronal activity with biologically grounded molecular context.

#### 2.4.1 Receptor-Enriched Analysis of Functional Connectivity by Targets (REACT)

**Figure 1:**
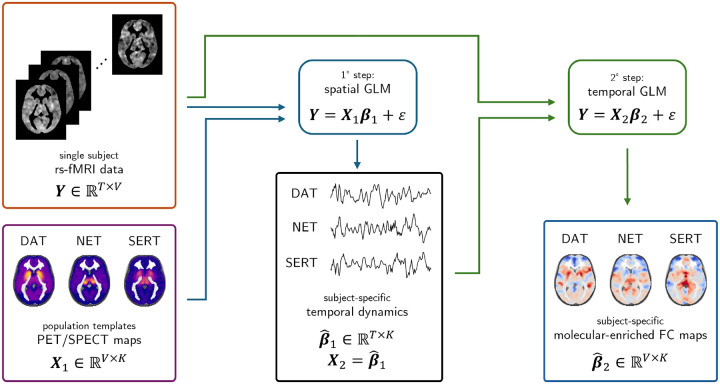
REACT workflow The REACT framework derives neurotransmitter-informed FC from rs-fMRI by combining subject BOLD data with population PET/SPECT receptor-density templates. Inputs are the BOLD matrix $Y\in {\mathbb{R}}^{T\times V}$ and the template matrix $X_{1}\in {\mathbb{R}}^{V\times K}$ (here, DAT, NET, and SERT). *Step 1 - spatial GLM: Y* = *X*_1_*β*_1_+ *ε* estimates, for each target, a subject-specific weight time series ${\hat{\beta }}_{1}\in {\mathbb{R}}^{T\times K}$ indexing how strongly that molecular distribution modulates ongoing BOLD fluctuations. *Step 2 - temporal GLM:* setting $X_{2}={\hat{\beta }}_{1},Y=X_{2}\beta_{2}+\varepsilon$ yields voxel-wise maps ${\hat{\beta }}_{2}\in {\mathbb{R}}^{V\times K}$ that represent receptor-enriched connectivity patterns.

The REACT framework enriches rs-fMRI BOLD fluctuations with molecular density data derived from publicly available PET/SPECT templates to derive neurotransmitter-informed FC maps (Dipasquale et al., 2019). It proceeds in two steps as depicted in Figure 1: a) spatial regression: each preprocessed rs-fMRI run is regressed in time onto a receptor-density template, yielding a subject- and target-specific weight time series that quantifies how strongly that molecular distribution modulates ongoing BOLD fluctuations; b) temporal regression: this weight time series is then entered as a regressor in space against the voxel-wise BOLD signal, producing a voxel-wise connectivity map enriched for the same receptor target, and thus revealing neurochemically informed circuit fingerprints.

#### 2.4.2 Neuroreceptor templates

Three templates for the transporters of dopamine (DAT), norepinephrine (NET), and serotonin (SERT) were selected. The DAT map was constructed from [^123^I]-ioflupane SPECT images acquired in 30 neurologically healthy adults showing no signs of nigrostriatal impairment (García-Gómez et al., 2013). The NET template was generated by averaging individual [^11^C]-MRB PET parametric maps from a cohort of 10 healthy subjects (Hesse et al., 2017). The SERT template was derived by pooling [^11^C]-DASB PET images from 210 healthy volunteers to generate a high-resolution binding distribution (Beliveau et al., 2017). All three templates were normalized into MNI152NLin6Asym space with 2 mm isotropic voxels, and scaled between 0 and 1.

These targets were selected a priori because monoaminergic transporters exhibit well-characterized age-related alterations and regulate large-scale network dynamics detectable with BOLD functional connectivity (Buchert et al., 2006; Ding et al., 2010; Kanel et al., 2023; Karrer et al., 2017). Dopaminergic markers, including transporter and receptor availability, decline across adulthood and relate to individual differences in cognitive performance (Karrer et al., 2017). Noradrenergic integrity, encompassing the locus coeruleus and its cortical projections, shows age-related change with established links to attention and arousal, and PET studies indicate reduced NET binding with age (Ding et al., 2010; Mather & Harley, 2016). Serotonergic terminals and transporter availability also display regional age-related reductions (Buchert et al., 2006; Kanel et al., 2023). All together, DAT, NET, and SERT provide biologically interpretable axes for molecular-enriched FC in brain-age models (Hansen et al., 2022). Although additional neurotransmitter systems and receptor subtypes also show age-dependent variation, we restricted the present analysis to monoaminergic transporters for methodological and comparability reasons: first, to maintain continuity with prior REACT applications and second, to limit spatial collinearity among molecular maps, particularly that arising between receptor subtypes with overlapping distributions, which can destabilize the two-stage regression and hinder interpretability (Beliveau et al., 2017; Dipasquale et al., 2019; Hansen et al., 2022).

#### 2.4.3 REACT implementation

The react-fmri tool (https://github.com/ottaviadipasquale/react-fmri) requires two binary masks for its two-stage regression: i) a Stage-1 mask restricting the temporal regression to voxels common to each transporter template and to cortical gray matter (GM); ii) a Stage-2 mask for the spatial regression, here taken as the GM mask alone. We generated the Stage-1 mask as the intersection between each transporter template and the generic GM mask supplied with REACT, and the Stage-2 mask as that GM mask alone.

Processing then proceeded in two steps. i) Each participant’s preprocessed BOLD data were processed with the command-line utility react-fmri, once per transporter template, yielding subject- and target-specific weight images and transporter-enriched functional-connectivity maps in MNI152NLin6Asym space. ii) Every transporter-enriched connectivity map was parcellated into 247 regions of interest (ROIs) — 200 cortical parcels from the Schaefer 7-network atlas (Schaefer et al., 2017), 15 subcortical regions from the Harvard–Oxford structural atlas (FMRIB Analysis Group, University of Oxford & Harvard Center for Morphometric Analysis, 2023; Smith et al., 2004), and 32 cerebellar regions from the SUIT atlas (Diedrichsen, 2009) — and the resulting #subjects×247 feature matrices (one per transporter) were exported as tab-separated value files for downstream brain-age modeling.

### 2.5 Brain-age modeling workflow

The preprocessing stage yielded, for every dataset and every transmitter system, a #subjects×247 feature matrix that served as input to a machine-learning pipeline for estimating brain-age.

**Feature blocks and models comparison**. Across all analyses we considered six model configurations: three transmitter-specific models (DAT, NET, SERT), each using 247 molecular-enriched FC regional features (mean receptor-enriched connectivity within each of the 247 ROIs defined above); a concatenated molecular-enriched features (MEF) block that stacks the three transmitter sets for a total of 3×247=741 features; a structural morphometry block (SMF, 649 features) comprising (a) 85 subcortical/global volumetric indices (Fischl et al., 2002), (b) 2×31 cortical regions × 8 metrics (GM volume, cortical surface area, mean cortical thickness, thickness standard deviation, folding index, mean curvature, Gaussian curvature, curvature index) from the Desikan–Killiany-Tourville atlas (DKT) parcellation (Desikan et al., 2006), and (c) 68 regional WM volumes; and a multimodal union (MMF) obtained by concatenating MEF and SMF for a total of 741+649=1,390 features.

**Learning algorithm**. We used support vector regression (SVR) with a radial-basis-function kernel as a compromise between predictive accuracy and computational efficiency, consistent with earlier benchmarking (Pinamonti & Sammassimo et al., 2025). All analyses were executed in a Conda environment on a Slurm-managed cluster to ensure software consistency. Models predicted chronological age from the feature blocks above and were evaluated with 10-fold cross-validation repeated 10 times (folds stratified by age). Within each split, all predictors were scaled with RobustScaler (median/IQR; scikit-learn), fitted on the training fold only and applied to the test data. Hyper-parameters (C, *γ*, *ε*) were tuned by nested grid search within each training fold.

To structure the study, we organized three complementary analysis blocks: (i) *single-cohort models* to test whether molecular-enriched FC improves brain-age estimation within individual datasets; (ii) *merged-cohort models* to achieve sample sizes typical of structural brain-age work and to assess whether multi-site harmonization is required and what are its optimal settings; (iii) a *common-parcellation analysis* to isolate parcellation as a potential confound by aligning structural and functional features on the same atlas.

#### 2.5.1 Single-cohort models: testing the added value of molecular-enriched FC

To test whether molecular-enriched FC added predictive information for brain-age estimation, we evaluated six pre-specified model configurations within each dataset separately. These configurations spanned structure-only models, neurotransmitter-specific FC variants, and structure+functional combinations, all trained and validated under the same preprocessing, scaling, and nested cross-validation procedures described above.

#### 2.5.2 Merged-cohort models: assessing harmonization for multi-site integration

To determine whether batch correction was necessary and how best to implement it, we merged Cam-CAN, HCP-Aging, and NKI-RS to reach sample sizes typical of structural brain-age studies and compared models before and after harmonization.

We first trained models on non-harmonized data to quantify multi-site effects, and then repeated the analyses after neuroCombat correction (with and without empirical Bayes (EB)), using acquisition site as batch and preserving age and sex as covariates (Fortin et al., 2018). Two harmonization configurations were tested: (a) per-feature location/scale adjustment *without* EB pooling (no information borrowing across features), and (b) ComBat *with* EB pooling (Fortin et al., 2017; Johnson et al., 2007). For the latter, rather than estimating a single set of ComBat parameters across all features jointly, we first partitioned the merged feature matrix into homogeneous “subgroups” to respect measurement type and biological domain: for FreeSurfer structural features, we grouped variables derived from the same .stats file and of the same nature (volumes, surface areas, cortical folding or curvature); for functional connectivity, we grouped edges by neuroreceptor system (DAT, NET, SERT) and by a single atlas-defined parcel set (one of the seven Schaefer networks, the combined subcortical mask, or the cerebellar mask). We then ran neuroCombat separately on each subgroup, with the argument eb=True to invoke EB pooling for that subgroup only. This strategy fits distinct empirical-Bayes priors and hyper-parameters for each feature domain, so that borrowing of strength occurs only among features of the same type, preserving modality-specific biological variance while attenuating scanner- and site-related effects within each subgroup (Fortin et al., 2017; Johnson et al., 2007). After harmonization, the corrected sub-matrices were recombined for downstream cross-validation and SVR modeling. For every harmonization setting, we retrained transmitter-specific models, the concatenated functional model, the FreeSurfer structural model, and the full multimodal model, following the same cross-validation protocol.

#### 2.5.3 Common-parcellation analysis: testing parcellation effects on multimodal gains

To isolate atlas mismatch as a potential confound in the merged-cohort setting, we projected both structural and neurotransmitter-weighted FC features onto identical subject-specific DKT parcels and retrained transmitter-specific, structural, and combined models after neuroCombat harmonization (no EB). This analysis tested whether molecular-enriched FC contributed information beyond FreeSurfer-derived structure when both were summarized on the same atlas.

#### 2.5.4 Evaluation metrics

Model accuracy was quantified with five complementary statistics computed in every test fold, following the evaluation framework proposed by Baecker et al. (2021). MAE measured the average unsigned deviation between predicted and chronological age, whereas root-mean-squared error (RMSE) penalized larger residuals by squaring them before averaging and taking the square root. Pearson’s correlation coefficient (*r*) captured the linear association between true and estimated ages, and the prediction coefficient of determination (*R*^2^) summarized the proportion of age variance explained in unseen data. Bias was assessed as Spearman’s rank correlation (*ρ*) between chronological age and the prediction error $\Delta \text{age}=\hat{\text{age}}-\text{age}$; values near zero indicate that errors are not systematically age-dependent.

## 3 Results

### 3.1 Datasets

Following MRIQC screening, motion censoring, and preprocessing, we excluded 226 participants, leaving 2,128 adults eligible for analysis across the three source studies. Because the HCP-Aging cohort contained only eight participants older than 90 years, too few for robust model estimation at that extreme age range, we excluded those individuals. The final analytic sample therefore comprised 2,120 participants. Table 3 summarizes sample size, sex distribution, and age statistics for each dataset. Further information on participant exclusion after MRIQC and preprocessing is reported in the Supplementary Materials.

### 3.2 Qualitative characteristics of the molecular-enriched FC maps

**Figure 2:**
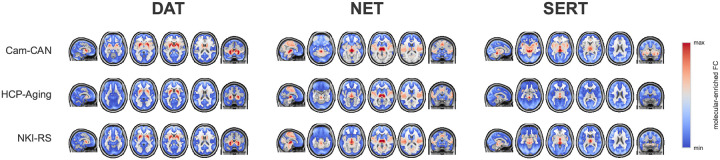
Group-mean molecular-enriched FC maps derived with REACT for the dopamine (DAT), norepinephrine (NET), and serotonin (SERT) transporters. Rows correspond to datasets (Cam–CAN, HCP–Aging, NKI–RS), columns to transporter targets. The color scale indicates relative connectivity (arbitrary units; min–max as shown). Consistent across datasets, DAT emphasizes anterior striatum/caudate head, NET emphasizes the cerebellar vermis–brainstem junction, and SERT emphasizes the midline cerebellar dentate/brainstem region, matching previously reported topographies (Cercignani et al., 2021; Coray et al., 2023; Dipasquale et al., 2023).

Figure 2 displays the group-mean transporter-enriched FC maps for each dataset (Cam-CAN, HCP-Aging, NKI-RS) and transmitter target (DAT, NET, SERT). Across cohorts, peak connectivity localizes to neuroanatomically plausible loci: the DAT map emphasizes the anterior striatum and caudate head; the NET map highlights the cerebellar vermis-brainstem junction; and the SERT map shows maximal signal in the midline cerebellar dentate/brainstem region. These spatial patterns align with prior reports of monoamine transporter–enriched connectivity (Cercignani et al., 2021; Coray et al., 2023; Dipasquale et al., 2023).

### 3.3 Brain-age modeling

The sections below report prediction accuracy and error metrics for the six model configurations including three individual neuroreceptor-enriched models, their combined model MEF, the structural model SMF and the multimodal union MMF.

#### 3.3.1 Single-cohort models: molecular-enriched FC generally adds predictive value

**Figure 3:**
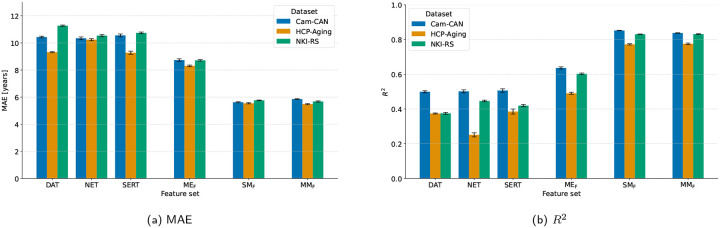
Comparison of model performance across feature sets (single transporter maps, molecular-enriched features (MEF), structural morphometric features (SMF), multimodal features (MMF)) between different datasets (Cam-CAN in blue, HCP-Aging in orange, NKI-RS in green): (a) mean absolute error (MAE) and (b) coefficient of determination (*R*^2^).

Table 4 first lists the accuracy obtained from the single transporter maps. Across cohorts, the DAT, NET, and SERT features yielded moderate explained variance in chronological age (*R*^2^ of 0.50–0.52 in Cam–CAN, 0.27–0.40 in HCP-Aging, 0.38–0.45 in NKI–RS), with MAEs clustered around 9.27–11.43 years. When the three functional maps were concatenated (MEF model), performance improved consistently, reducing the typical MAE by 0.96–1.82 years relative to the best single map and raising *R*^2^ into the 0.50–0.64 range. FreeSurfer-derived structural metrics (SMF model) remained the strongest single modality, posting the lowest MAEs (5.55–5.76 years) and the highest coefficients of determination (*R*^2^ of 0.78–0.85). Merging MEF with SMF (MMF model) retained this high accuracy and slightly surpassed pure structure in HCP-Aging (MAE = 5.49 vs. 5.55) and NKI-RS (MAE = 5.68 vs. 5.76). In summary, FS-derived structural features were most accurate in Cam–CAN, whereas the combined structural+functional set delivered the best result in HCP-Aging and NKI–RS. As illustrated in Figure 3, these numeric differences are echoed graphically: panels (a)–(b) show that MAE falls and *R*^2^ rises from the single-transporter maps (DAT, NET, SERT) to the concatenated REACT features, and again to the structural and multimodal models.

#### 3.3.2 Merged-cohort models: dataset harmonization is necessary

**Figure 4:**
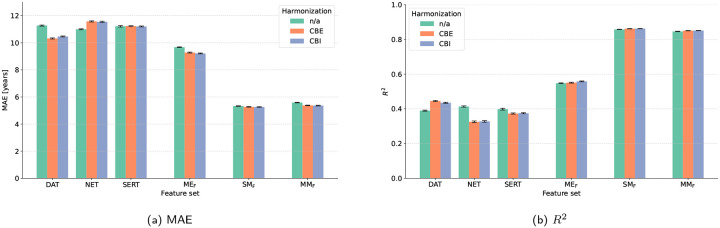
Comparison of model performance across feature sets for multi-site dataset and different harmonization strategies: (a) mean absolute error (MAE) and (b) coefficient of determination (*R*^2^).

Performance for the merged cohort is shown in Table 5. For the raw (non-harmonized) data, single-map functional inputs produced MAEs of 11.00–11.27 years; MEF concatenation lowered the error to 9.67 years; and FS structural features achieved 5.33 years, whereas the MMF model did not improve that result (MAE = 5.59). Applying ComBat with empirical-Bayes pooling (CBE) raised DAT and MEF accuracy slightly but did not help NET or SERT, while structural performance remained stable. The independent-feature ComBat configuration (CBI) delivered the overall best figures, marginally improving SMF (achieving *R*^2^ of 0.86 with MAE = 5.26 years), while MMF achieved the next-lowest error (MAE=5.36 years). Thus, SMF harmonized with independent-feature ComBat represented the top multi-site model, followed closely by the combined structural+functional feature set. The same pattern can be seen in Figure 4: harmonization improved performance overall, but did not reorder the hierarchy of the models.

#### 3.3.3 Common-parcellation analysis: parcellation mismatch explains the lost gains

**Figure 5:**
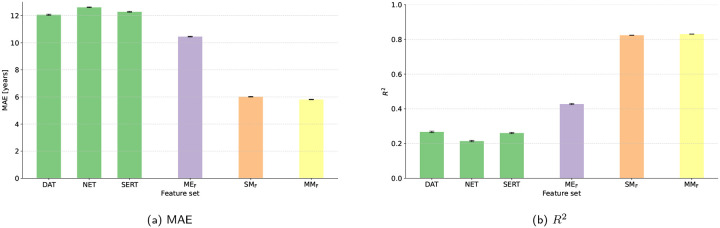
Comparison of model performance across feature sets on common brain parcellation: (a) mean absolute error (MAE) and (b) coefficient of determination (*R*^2^).

The DKT-based analysis (Table 6) on the merged cohort (CBI harmonization) shows similar trends when reanalyzed on a common parcellation. Single-map functional inputs produced MAEs of 12.06–12.61 years, while the MEF concatenation reduced error to 10.45 years. SMF alone achieved an MAE of 6.02 years, and combining DKT structure with the three functional maps (MMF) lowered the error slightly further to 5.81 years with *R*^2^ of 0.83; compared to SMF, the absolute gain in performance is 0.21 years (3.5% improvement). These effects are visualized in Figure 5, which confirms that the joint DKT structural+functional model yields the lowest MAE and highest *R*^2^ within this parcellation framework.

## 4 Discussion

We asked whether molecularly informed resting-state FC derived with REACT added predictive value to structural MRI for brain-age estimation. Using three adult cohorts (Cam–CAN, HCP–Aging, and NKI–RS), we trained SVR models on transporter-enriched maps (DAT, NET and SERT), FS morphometry, and their combination, and systematically assessed multi-site harmonization with ComBat. We then enforced a common parcellation to compute parcel-level features from both T1w and rs-fMRI modalities within the same anatomical node space, enabling direct cross-modality comparisons.

Across 2,120 adults, molecular-enriched FC was predictive of brain-age and, when added to morphometry, provided incremental value over a structure-only baseline. Within single cohorts, aggregating the three transmitter-weighted maps into one molecular-enriched FC block increased explained variance over any single transmitter map and, when added to FreeSurfer morphometry, yielded small, cohort-dependent gains. After merging datasets, harmonization was necessary to preserve the predictive value of molecular-enriched FC, with the independent-feature ComBat configuration (CBI) performing best and maintaining the multimodal advantage. Aligning modalities to a common atlas (DKT) restored a consistent improvement on the merged cohort, implicating parcellation mismatch as the principal reason gains appeared attenuated without alignment.

### 4.1 Brain-age with molecular-enriched FC

#### 4.1.1 Single-cohort models: cohort-dependent gains

Molecular-enriched FC alone predicted chronological age with moderate explained variance, and concatenating the three transmitter weightings reliably boosted *R*^2^ relative to any single map. Adding molecular-enriched FC to morphometry further improved performance in two cohorts (HCP–Aging and NKI–RS) while showing no gain in Cam–CAN. A minimal set of error metrics was consistent with this view: single-transmitter models had MAE on the order of 9–11 years, and concatenation typically reduced MAE by roughly 1–2 years per cohort, mirroring the rise in *R*^2^. The relative ranking of transmitter-specific maps varied by dataset. SERT/DAT performed slightly better than NET in Cam–CAN and HCP–Aging, whereas NET led in NKI–RS, suggesting cohort- and acquisition-dependent sensitivity of neuromodulatory circuits to aging. These findings indicate that transmitter-anchored connectivity captures unique age-related variance and yields a measurable, albeit cohort- and acquisition-dependent, incremental gain. Structural metrics remain a strong baseline, but molecular-enriched FC is itself predictive and can improve estimates.

#### 4.1.2 Merged-cohort models: harmonization is needed

Pooling Cam–CAN, HCP–Aging, and NKI–RS introduced site-related shifts that disproportionately affected functional features. Harmonization was essential to preserve and express the predictive value of molecular-enriched FC. Among tested strategies, CBI provided the best balance, recovering the strongest molecular-enriched FC block while maintaining high structural performance and the expected multimodal advantage. In line with the single-cohort trends, CBI modestly lowered error for molecular-enriched FC (e.g., from MAE 9.67 to 9.21 years for the concatenated block) and set a reference point for structure (5.26 years) without altering the qualitative hierarchy of feature sets. Taken together, these observations motivate a practical recommendation: deploy domain-specific pooling of harmonization parameters via CBI in multi-site brain-age pipelines, as it curbs scanner/site effects that otherwise degrade features.

#### 4.1.3 Common-parcellation analysis: atlas alignment restores molecular-enriched FC gains

Because functional gains appeared smaller after merging datasets, we examined whether parcellation mismatch masked true complementarity. Enforcing a shared cortical atlas (DKT) for both modalities clarified the picture: adding molecular-enriched FC to structure consistently improved prediction on the merged cohort, raising *R*^2^ from 0.824 to 0.830 with a parallel error reduction (from MAE 6.02 to 5.81 years). Thus, when node definitions are aligned, the incremental contribution of molecular-enriched FC is expressed reliably, and the earlier attenuation is best attributed to representational mismatch rather than weak functional signal.

In summary, these results converge on a coherent interpretation: molecular-enriched FC carries age-related signal that is not redundant with morphometry and, under routine multi-site constraints, can be translated into measurable improvements. Within single cohorts, concatenated transmitter weightings increase *R*^2^ and modestly reduce MAE; across cohorts, those gains are preserved when harmonization adopts CBI; and when modalities share the same parcellation, the multimodal advantage reappears consistently. In practice, treating molecular-enriched FC as a first-class feature block, paired with independent-feature ComBat and atlas alignment, yields brain-age models that retain mechanistic interpretability and, depending on cohort and alignment, match or surpass structure-only baselines.

### 4.2 Comparison with literature

Our findings are consistent with recent brain-age reports in both healthy and clinical cohorts. Using only T1w MRI, we achieved a MAE of roughly 5–6 years, matching the performance range described by Cole and Franke (2017). A study by Liem et al. (2017), which analyzed a slightly larger cohort of more than 2,300 adults and combined cortical thickness with whole-brain rs-FC features, reported a MAE of 4.29 years. In comparison, our best multimodal model, trained on about 2,100 participants and integrating FreeSurfer morphometry with molecular-enriched FC, reached a MAE of 5.36 years. The small gap likely reflects differences in sample composition and the added variability introduced by pooling data across scanners. Importantly, our SMF model attained an *R*^2^ near 0.85, a level comparable to those single-site studies.

Liem et al. (2017) also observed that adding FC to structural features improved accuracy. In our data, structural metrics were already highly predictive, so the molecular-enriched FC features provided only a modest additional benefit; nonetheless, they did lower the residual error and increased explained variance, confirming that age-related changes in functional networks convey information not captured by anatomy alone. This discrepancy may be due to differences in functional feature richness: Liem et al. (2017) used full resting-state connectivity matrices, which encompass aging effects on diverse networks, whereas we focused on specific neurotransmitter-enriched connections. It is plausible that our functional features, being constrained to DAT-, NET-, and SERT-related circuits, captured a narrower slice of age-related functional change, thus emphasizing neurochemical network effects but potentially missing other widespread changes (e.g., in default-mode or visual networks) that whole-brain FC approaches exploit. Future studies could explore augmenting our molecular connectivity features with broader functional measures to see whether multimodal combinations can surpass structure-only models, as reported by Liem et al. (2017) and others.

Recent work has indeed demonstrated that resting-state FC alone can be a potent predictor of age. Bi et al. (2024) used the Cam–CAN dataset and reported that both movie-watching and resting-state FC yielded high correlation with age (*R*^2^=0.74). This accuracy is comparable to, and in some cases exceeds, the performance of structural MRI models. Our results temper this optimistic view by showing that, in a direct comparison within the same individuals, structural features still provided substantially higher *R*^2^ (up to 0.85) than any single functional feature (*R*^2^=0.51 at best), and even the multi-network FC model (MEF) reached only *R*^2^=0.64 (Table 4). It is important to note that Bi et al. (2024) and similar studies typically employ many more FC features (e.g., thousands of connections) and often use advanced machine-learning or deep-learning models that can capture non-linear aging patterns in connectivity. In contrast, our approach emphasizes interpretability by using predefined molecular circuits and a relatively simple SVR model. The trade-off is a lower ceiling on FC-based accuracy, but with the benefit of linking age effects to specific neurochemical systems. Encouragingly, our MEF model’s MAEs (8.31–8.72 years single-site, 9.21 years multi-site) are on par with other moderate-sized FC studies (Gonneaud et al., 2021; Millar et al., 2022; Zhai & Li, 2019), and with further refinement these numbers could improve.

Our work also connects with emerging findings in pathological aging. Millar et al. (2022) examined brain-age in preclinical and symptomatic Alzheimer’s disease (AD) using functional and structural MRI. They found that an FC-only age model detected aberrant brain aging in asymptomatic individuals at risk for AD, even when structural MRI appeared normal. In our healthy dataset we do not directly test disease effects, but the strong performance of structural features implies they capture the lion’s share of normative aging variance, whereas FC may capture subtler, system-specific changes that could represent early signs of neurodegeneration before structural atrophy becomes evident. Intriguingly, Millar et al. (2022) reported that while both functional and structural brain-age gap metrics were elevated in cognitively impaired AD patients, only the FC-based brain age showed a paradoxical decrease (younger-appearing brains) in the preclinical stage, possibly reflecting transient network reorganization. Such complex, bi-phasic effects underscore that FC and structural measures are complementary: FC might be more sensitive to early or compensatory changes, whereas structural changes correlate more linearly with cumulative damage.

Although we did not find a major boost in accuracy from adding FC in healthy adults, it is likely that in a clinical context the functional features would add important diagnostic value, e.g., flagging individuals whose brain connectivity appears “older” or “younger” than expected for their age in ways that structural MRI cannot. In the literature, several multimodal brain-age studies (e.g., Liem et al., 2017; Millar et al., 2023) conclude that integrating across modalities yields the most robust predictors and better captures cognitive or pathological deviations. To our knowledge, this is the first large-scale, multi-site brain-age investigation to incorporate molecular-enriched FC, adding a molecularly informed functional dimension to brain-age estimation. Future comparisons with other multimodal frameworks will clarify optimal strategies for fusing anatomical, functional, and molecular imaging data.

### 4.3 Limitations

This study nonetheless has a few noteworthy limitations. First, although we used a non-linear support-vector regression with a radial-basis-function kernel, SVR remains constrained by pair-wise similarity structure and a relatively small hyper-parameter set, which can limit its ability to model complex feature interactions. In line with this, our prior benchmarking across kernel-based and ensemble methods showed that model choice and performance depend strongly on data distribution and harmonization: gradient-boosted trees (XGB) were most robust on non-harmonized data, whereas SVR achieved the best accuracy after ComBat (Pinamonti & Sammassimo et al., 2025). These results suggest that no single algorithm is uniformly optimal and motivate exploration of larger training sets and higher-capacity models (e.g., ensembles or deep learning), while acknowledging trade-offs in interpretability and over-fitting risk. Second, we extracted atlas-level features and compared results across two different parcellations. Alternative parcellations, or voxel-wise, surface-based, or graph-theoretic representations, could reveal finer-grained or topological aging effects, particularly in functional connectivity. Third, our multi-site harmonization relied on ComBat. While overall performance improved, effects were uneven and transmitter-dependent: NET deteriorated under both harmonization variants and SERT remained essentially unchanged. These patterns suggest possible over-correction for some features or residual entanglement between site and age effects; future work could explore site-invariant representation learning or hierarchical models. Fourth, the study is cross-sectional. Without longitudinal follow-up we cannot determine whether participants with higher brain-age gaps actually age faster. Longitudinal validation and uncertainty quantification are essential for clinical deployment, where single-subject decisions require tighter error bounds than the 5-years MAE achieved here. Fifth, molecular enrichment was based on population-average PET/SPECT templates for only three monoaminergic transporters. Inter-individual variability in receptor density and aging effects in other neurotransmitter systems were not modeled. Incorporating subject-specific molecular imaging or additional receptor atlases could enhance sensitivity. Finally, our analytic sample comprised healthy adults from Cam-CAN (UK), HCP-Aging (USA), and NKI-RS (USA), screened as cognitively normal according to each cohort’s original protocols. Generalizability to other demographic, socioeconomic, or clinical populations remains to be established and should be tested in more diverse, longitudinal cohorts.

## 5 Conclusion

Our goal was to advance brain-age modeling by pairing established morphometric predictors with novel, molecular-enriched FC features and to test their performance across multiple imaging sites. Consistent with our hypothesis, structural measures yielded the highest standalone accuracy, with MAE in the five-to-six-year range and excellent generalizability across scanners.

Interestingly, the inclusion of neurotransmitter-weighted FC maps into a brain-age model explained a significant amount of brain age variance that structure alone did not capture. When DAT, NET, and SERT-enriched networks were added to the model, prediction error decreased, indicating that functional alterations within monoaminergic circuits contribute complementary aging-related information beyond structural anatomy. These gains, though modest in absolute terms, were consistent across atlases and remained after ComBat harmonization, underscoring that neuromodulatory connectivity holds independent predictive value beyond macroscopic atrophy.

The findings therefore highlight two complementary facets of cerebral aging: widespread tissue loss, which drives the bulk of structural prediction, and systematic alterations in neurochemical functional networks, which refine, and potentially re-interpret, brain-age estimates. Future work should deepen this molecular perspective by integrating a broader repertoire of receptor systems, employing individual PET/SPECT maps where available, and examining longitudinal or clinical cohorts in whom neuromodulatory dysfunction may emerge before overt atrophy. Such efforts could transform molecular-enriched FC from a modest performance booster into a sensitive early marker that enriches the biological meaning and clinical utility of brain-age metrics.

## Supplementary Material

1

## Figures and Tables

**Table 1:** T1-weighted structural MRI acquisition parameters for each cohort

Dataset	Sequence	TR [ms]	TE [ms]	TI [ms]	FA [°]	FOV [mm]	Voxel size [mm]
Cam-CAN	MPRAGE	2250	2.99	900	9	256×240×192	1×1×1
HCP-Aging	ME MPRAGE	2500	1.8/3.6/5.4/7.2	1000	8	256×240×166	0.8×0.8×0.8
NKI-RS	MPRAGE	1900	2.52	900	9	256×246×176	1×1×1

MPRAGE = Magnetization Prepared Rapid Gradient Echo; ME = multi-echo; TR = repetition time; TE = echo time (for HCP-Aging, four echoes); TI = inversion time; FA = flip angle; FOV = field of view.

**Table 2:** Resting–state functional MRI acquisition parameters for each cohort

Dataset	Sequence	TR [ms]	TE [ms]	FA [°]	FOV [mm]	Voxel size [mm]	Slices	Volumes	Time [min:sec]
Cam-CAN	SB GE-EPI	1970	30	78	192×192	3×3×4.4	32	261	8:34
HCP-Aging	MB8 GE-EPI	870	37	52	208×208	2×2×2	72	488	7:05
NKI-RS	MB4 GE-EPI	1400	30	65	224×224	2×2×2	64	418	9:45

SB = single-band; MB = multi-band; GE = gradient-echo; EPI = echo-planar imaging; TR = repetition time; TE = echo time; TI = inversion time; FA = flip angle; FOV = field of view.

**Table 3:** Demographic characteristics of the quality-controlled cohorts.

Dataset	*N*	Female / Male	Age, mean ± SD [years]	Age range [years]
Cam-CAN	627	315 / 312	53.8 ± 18.5	18–88
HCP-Aging	599	337 / 262	58.6 ± 14.7	36–90
NKI-RS	894	591 / 303	46.7 ± 17.7	18–85
**Total**	**2,120**	**1,243 / 877**	52.2 ± 17.9	18–90

**Table 4:** Single-site brain-age prediction performance across feature sets

Feature	Dataset	*r*	*R* ^2^	MAE [years]	RMSE [years]	*ρ*
DAT	Cam-CAN	0.713 ± 0.006	0.499 ± 0.009	10.432 ± 0.090	13.032 ± 0.115	−0.682 ± 0.013
	HCP-Aging	0.621 ± 0.004	0.374 ± 0.004	9.315 ± 0.049	11.604 ± 0.054	−0.734 ± 0.005
	NKI-RS	0.618 ± 0.005	0.375 ± 0.007	11.273 ± 0.064	13.930 ± 0.088	−0.750 ± 0.006
NET	Cam-CAN	0.713 ± 0.007	0.502 ± 0.012	10.342 ± 0.138	13.002 ± 0.158	−0.661 ± 0.007
	**HCP-Aging**	0.519 ± 0.015	0.251 ± 0.017	10.239 ± 0.106	12.714 ± 0.135	−0.805 ± 0.010
	NKI-RS	0.673 ± 0.005	0.446 ± 0.007	10.540 ± 0.114	13.116 ± 0.083	−0.701 ± 0.009
SERT	Cam-CAN	0.718 ± 0.009	0.506 ± 0.014	10.555 ± 0.147	12.935 ± 0.177	−0.654 ± 0.013
	**HCP-Aging**	**0.628** ± 0.014	**0.385** ± 0.020	**9.274** ± 0.161	**11.511** ± 0.195	−**0.734** ± 0.011
	NKI-RS	0.653 ± 0.006	0.419 ± 0.009	10.740 ± 0.088	13.421 ± 0.105	−0.709 ± 0.004
MEF	Cam-CAN	0.802 ± 0.006	0.636 ± 0.009	8.734 ± 0.136	11.100 ± 0.155	−0.558 ± 0.011
	**HCP-Aging**	**0.707** ± 0.006	**0.491** ± 0.009	**8.311** ± 0.077	**10.466** ± 0.089	−**0.665** ± 0.014
	NKI-RS	0.780 ± 0.004	0.603 ± 0.006	8.717 ± 0.081	11.100 ± 0.078	−0.607 ± 0.007
SMF	Cam-CAN	0.925 ± 0.001	0.852 ± 0.002	5.623 ± 0.052	7.073 ± 0.039	−0.372 ± 0.011
	**HCP-Aging**	**0.882** ± 0.003	**0.772** ± 0.005	**5.547** ± 0.064	**7.000** ± 0.077	−**0.470** ± 0.011
	NKI-RS	0.913 ± 0.001	0.831 ± 0.002	5.763 ± 0.033	7.242 ± 0.041	−0.410 ± 0.011
MMF	Cam-CAN	0.917 ± 0.002	0.838 ± 0.002	5.858 ± 0.042	7.399 ± 0.052	−0.385 ± 0.011
	**HCP-Aging**	**0.884** ± 0.004	**0.776** ± 0.006	**5.485** ± 0.059	**6.939** ± 0.092	−**0.480** ± 0.010
	NKI-RS	0.913 ± 0.002	0.831 ± 0.003	5.679 ± 0.062	7.222 ± 0.067	−0.420 ± 0.008

Metrics reported: *r* (Pearson’s correlation coefficient), *R*^2^ (coefficient of determination), MAE (mean absolute error), RMSE (root mean square error), *ρ* (age-bias correlation). Each value is the mean (averaged over the 10 folds and 10 repetitions) ± standard deviation (SD) (computed across the 10 repetitions only) from a 10 × 10-fold cross-validation (CV), for a total of 100 trained models per configuration.

**Table 5:** Multi-site brain-age prediction performance across harmonization procedures

Harmonization	Feature	*r*	*R* ^2^	MAE [years]	RMSE [years]	*ρ*
n/a	DAT	0.630 ± 0.003	0.389 ± 0.004	11.271 ± 0.053	13.935 ± 0.053	−0.727 ± 0.003
	NET	0.647 ± 0.004	0.413 ± 0.005	10.997 ± 0.056	13.666 ± 0.063	−0.724 ± 0.007
	SERT	0.640 ± 0.005	0.398 ± 0.007	11.218 ± 0.077	13.839 ± 0.085	−0.704 ± 0.007
	MEF	0.743 ± 0.002	0.548 ± 0.003	9.674 ± 0.035	11.996 ± 0.039	−0.640 ± 0.004
	**SMF**	**0.927** ± 0.001	**0.858** ± 0.001	**5.332** ± 0.029	**6.728** ± 0.034	−**0.357** ± 0.004
	MMF	0.920 ± 0.001	0.846 ± 0.002	5.591 ± 0.033	7.003 ± 0.041	−0.384 ± 0.006
CBE	DAT	0.669 ± 0.004	0.445 ± 0.005	10.320 ± 0.056	13.283 ± 0.057	−0.714 ± 0.006
	NET	0.577 ± 0.004	0.325 ± 0.007	11.576 ± 0.059	14.653 ± 0.071	−0.772 ± 0.005
	SERT	0.615 ± 0.003	0.373 ± 0.005	11.232 ± 0.049	14.129 ± 0.055	−0.740 ± 0.004
	MEF	0.743 ± 0.003	0.551 ± 0.004	9.278 ± 0.051	11.955 ± 0.052	−0.664 ± 0.005
	**SMF**	**0.929** ± 0.001	**0.862** ± 0.001	**5.273** ± 0.033	**6.632** ± 0.033	−**0.351** ± 0.006
	MMF	0.923 ± 0.001	0.850 ± 0.002	5.375 ± 0.036	6.897 ± 0.036	−0.394 ± 0.004
CBI	DAT	0.662 ± 0.003	0.435 ± 0.004	10.468 ± 0.051	13.406 ± 0.054	−0.715 ± 0.003
	NET	0.579 ± 0.004	0.328 ± 0.007	11.549 ± 0.061	14.626 ± 0.072	−0.771 ± 0.005
	SERT	0.617 ± 0.004	0.375 ± 0.005	11.206 ± 0.050	14.108 ± 0.055	−0.738 ± 0.004
	MEF	0.748 ± 0.002	0.558 ± 0.004	9.212 ± 0.045	11.855 ± 0.049	−0.660 ± 0.005
	**SMF**	**0.929** ± 0.001	**0.862** ± 0.001	**5.263** ± 0.034	**6.619** ± 0.033	−**0.348** ± 0.005
	MMF	0.923 ± 0.001	0.851 ± 0.002	5.364 ± 0.036	6.882 ± 0.035	−0.394 ± 0.004

Metrics reported: *r* (Pearson’s correlation coefficient), *R*^2^ (coefficient of determination), MAE (mean absolute error), RMSE (root mean square error), *ρ* (age-bias correlation). Each value is the mean (averaged over the 10 folds and 10 repetitions) ± SD (computed across the 10 repetitions only) from a 10 × 10-fold CV, for a total of 100 trained models per configuration.

**Table 6:** Multi-site brain-age prediction performance on common brain parcellation

Feature	*r*	*R* ^2^	MAE [years]	RMSE [years]	*ρ*
DAT	0.532 ± 0.005	0.267 ± 0.006	12.064 ± 0.063	15.272 ± 0.065	−0.774 ± 0.006
NET	0.477 ± 0.004	0.214 ± 0.006	12.610 ± 0.039	15.814 ± 0.062	−0.828 ± 0.005
SERT	0.517 ± 0.004	0.260 ± 0.006	12.281 ± 0.043	15.344 ± 0.060	−0.811 ± 0.005
MEF	0.657 ± 0.003	0.427 ± 0.005	10.453 ± 0.039	13.494 ± 0.058	−0.707 ± 0.004
SMF	0.908 ± 0.001	0.824 ± 0.001	6.017 ± 0.031	7.489 ± 0.024	−0.400 ± 0.004
**MMF**	**0.912** ± 0.000	**0.830** ± 0.001	**5.809** ± 0.027	**7.343** ± 0.023	−**0.398** ± 0.002

Metrics reported: *r* (Pearson’s correlation coefficient), *R*^2^ (coefficient of determination), MAE (mean absolute error), RMSE (root mean square error), *ρ* (age-bias correlation). Each value is the mean (averaged over the 10 folds and 10 repetitions) ± SD (computed across the 10 repetitions only) from a 10 × 10-fold CV, for a total of 100 trained models per configuration.

## Data Availability

All neuroimaging data used in this study are publicly accessible. T1w and rs-fMRI scans from Cam-CAN are available at https://camcan-archive.mrc-cbu.cam.ac.uk/dataaccess/ (Shafto et al., 2014; Taylor et al., 2017). HCP-Aging data can be downloaded from https://www.humanconnectome.org/study/hcp-lifespan-aging (Bookheimer et al., 2019; Harms et al., 2018). NKI-RS data are provided at https://fcon_1000.projects.nitrc.org/indi/enhanced/ (Nooner et al., 2012; Tobe et al., 2022). Preprocessing pipelines relied exclusively on publicly available BIDS-Apps: functional MRI Preprocessing (fMRIPrep) (v23.2.2, https://fmriprep.org/en/23.2.2/), XCP-D (v0.7.4, https://xcp-d.readthedocs.io/en/0.7.4/), and MRIQC (v24.1.0, https://mriqc.readthedocs.io/en/latest/) or common neuroimaging tools as FSL (v7.3.2, https://fsl.fmrib.ox.ac.uk/fsl/docs/). REACT was implemented with the open-source react-fmri package, available at https://github.com/ottaviadipasquale/react-fmri. Custom scripts for brain-age modeling (SVR) and feature harmonization with ComBat were written for local execution and are available upon reasonable request from the corresponding author under a standard data-use agreement.

## Footnotes

1 https://github.com/bids-standard/bids-validator, https://bids-validator.readthedocs.io/en/latest/

2 https://github.com/nipreps/mriqc, https://mriqc.readthedocs.io/

## References

[R1] Alzheimer’s Disease International. (2024). World Alzheimer report 2024. https://www.alzint.org/resource/world-alzheimer-report-2024/

[R2] Baecker L., Dafflon J., Reisert M., Stüber C., Wiest R., Reyes M., (2021). Brain age prediction: A comparison between machine learning models using region- and voxel-based morphometric data. Human Brain Mapping, 42, 2332–2346. 10.1002/hbm.25368 33738883 PMC8090783

[R3] Behzadi Y., Restom K., Liau J., & Liu T. T. (2007). A component based noise correction method (CompCor) for BOLD and perfusion based fMRI. NeuroImage, 37(1), 90–101. 10.1016/j.neuroimage.2007.04.042 17560126 PMC2214855

[R4] Beliveau V., Ganz M., Feng L., Ozenne B., Højgaard L., Fisher P. M., Svarer C., Greve D. N., & Knudsen G. M. (2017). A high-resolution in vivo atlas of the human brain’s serotonin system. Journal of Neuroscience, 37(1), 120–128. 10.1523/jneurosci.2830-16.2016 28053035 PMC5214625

[R5] Betzel R. F., Byrge L., He Y., Goñi J., Zuo X.-N., & Sporns O. (2014). Changes in structural and functional connectivity among resting-state networks across the human lifespan. NeuroImage, 102(Part 2), 345–357. 10.1016/j.neuroimage.2014.07.067 25109530

[R6] Bi S., Guan Y., & Tian L. (2024). Prediction of individual brain age using movie and resting-state fMRI. Cerebral Cortex, 34(1), bhad407. 10.1093/cercor/bhad407 37885127

[R7] BIDS Apps Community. (2025). Bids apps: Portable neuroimaging pipelines. https://bids-apps.neuroimaging.io/

[R8] Blair R., Michael Z., Gorgolewski K. J., Hardcastle N., Hobson-Lowther T., Nishikawa D., Bhogawar S., Appelhoff S., Jas M., Grass B., Markiewicz C. J., Holdgraf C., Jones A., Goyal R., Oostenveld R., Noack G., Triplett W., Naveau M., Zito M., … Zulfikar W. (2024). Bidsvalidator. Zenodo. 10.5281/zenodo.3688706

[R9] Blinkouskaya Y., Caçoilo A., Gollamudi T., Jalalian S., & Weickenmeier J. (2021). Brain aging mechanisms with mechanical manifestations. Mechanisms of Ageing and Development, 200, 111575. 10.1016/j.mad.2021.111575 34600936 PMC8627478

[R10] Bookheimer S. Y., Salat D. H., Terpstra M., Ances B. M., Barch D. M., Buckner R. L., Burgess G. C., Curtiss S. W., Diaz-Santos M., Elam J. S., Fischl B., Greve D. N., Hagy H. A., Harms M. P., Hatch O. M., Hedden T., Hodge C., Japardi K. C., Kuhn T. P., … Yacoub E. (2019). The lifespan Human Connectome Project in aging: An overview. NeuroImage, 185, 335–348. 10.1016/j.neuroimage.2018.10.009 30332613 PMC6649668

[R11] Botvinik-Nezer R., Iwanir R., Holzmeister F., Huber J., Johannesson M., Kirchler M., Dreber A., Camerer C. F., Poldrack R. A., & Schonberg T. (2019). fMRI data of mixed gambles from the neuroimaging analysis replication and prediction study. Scientific Data, 6, 106. 10.1038/s41597-019-0113-7 31263104 PMC6602933

[R12] Bowring A., Maumet C., & Nichols T. E. (2019). Exploring the impact of analysis software on task fMRI results. Human Brain Mapping, 40, 3362–3384. 10.1002/hbm.24603 31050106 PMC6618324

[R13] Brusini I., MacNicol E., Kim E., Smedby Ö., Wang C., Westman E., Veronese M., Turkheimer F., & Cash D. (2022). MRI-derived brain age as a biomarker of ageing in rats: Validation using a healthy lifestyle intervention. Neurobiology of Aging, 109, 204–215. 10.1016/j.neurobiolaging.2021.10.004 34775211

[R14] Buchert R., Schulze O., Wilke F., Berding G., Thomasius R., Petersen K., Brenner W., & Clausen M. (2006). Is correction for age necessary in SPECT or PET of the central serotonin transporter in young, healthy adults? Journal of Nuclear Medicine, 47(1), 38–42. http://jnm.snmjournals.org/content/47/1/38.full.pdf 16391185

[R15] Cercignani M., Dipasquale O., Bogdan I., Carandini T., Scott J., Rashid W., Sabri O., Hesse S., Rullmann M., Lopiano L., Veronese M., Martins D., & Bozzali M. (2021). Cognitive fatigue in multiple sclerosis is associated with alterations in the functional connectivity of monoamine circuits. Brain Communications, 3(2), fcab023. 10.1093/braincomms/fcab023 PMC8023545 33842886

[R16] Chen Y., Liu Y. n., Zhou P., Zhang X., Wu Q., Zhao X., & Ming D. (2019). The transitions between dynamic micro-states reveal age-related functional network reorganization. Frontiers in Physiology, 9, 1852. 10.3389/fphys.2018.01852 30662409 PMC6328489

[R17] Ciric R., Rosen A. F. G., Erus G., Cieslak M., Adebimpe A., Cook P. A., Bassett D. S., Davatzikos C., Wolf D. H., & Satterthwaite T. D. (2018). Mitigating head-motion artifact in functional connectivity MRI. Nature Protocols, 13, 2801–2826. 10.1038/s41596-018-0065y 30446748 PMC8161527

[R18] Ciric R., Thompson W. H., Lorenz R., Goncalves M., MacNicol E. E., Markiewicz C. J., Halchenko Y. O., Ghosh S. S., Gorgolewski K. J., Poldrack R. A., & Esteban O. (2022). TemplateFlow: FAIR-sharing of multi-scale, multi-species brain models. Nature Methods, 19, 1568–1571. 10.1038/s41592-022-01681-2 36456786 PMC9718663

[R19] Cole J. H., Annus T., Wilson L. R., Remtulla R., Hong Y. T., Fryer T. D., Acosta-Cabronero J., Cardenas-Blanco A., Smith R., Menon D. K., Zaman S. H., Nestor P. J., & Holland A. J. (2017). Brain-predicted age in Down syndrome is associated with beta amyloid deposition and cognitive decline. Neurobiology of Aging, 56, 41–49. 10.1016/j.neurobiolaging.2017.04.006 28482213 PMC5476346

[R20] Cole J. H., & Franke K. (2017). Predicting age using neuroimaging: Innovative brain ageing biomarkers. Trends in Neurosciences, 40(12), 681–690. 10.1016/j.tins.2017.10.001 29074032

[R21] Cole J. H., Franke K., & Cherbuin N. (2019). Quantification of the biological age of the brain using neuroimaging. In Moskalev A. (Ed.), Biomarkers of human aging (pp. 293–328, Vol. 10). Springer. 10.1007/978-3-030-24970-0_19

[R22] Cole J. H., Leech R., Sharp D. J., & Alzheimer’s Disease Neuroimaging Initiative. (2015). Prediction of brain age suggests accelerated atrophy after traumatic brain injury. Annals of Neurology, 77(4), 571–581. 10.1002/ana.24367 25623048 PMC4403966

[R23] Cole J. H., Underwood J., Caan M. W. A., De Francesco D., van Zoest R. A., Leech R., Wit F. W. N. M., Portegies P., Geurtsen G. J., Schmand B. A., Schim van der Loeff M. F., Franceschi C., Sabin C. A., Majoie C. B., Winston A., Reiss P., & Sharp D. J. (2017). Increased brain-predicted aging in treated HIV disease. Neurology, 88(14), 1349–1357. 10.1212/wnl.0000000000003790 28258081 PMC5379929

[R24] Coray R. C., Zimmermann J., Haugg A., Baumgartner M. R., Steuer A. E., Seifritz E., Stock A.-K., Beste C., Cole D. M., & Quednow B. B. (2023). The functional connectome of 3,4-methyldioxymethamphetamine-related declarative memory impairments. Human Brain Mapping, 44(15), 5079–5094. 10.1002/hbm.26438 37530403 PMC10502674

[R25] Desikan R. S., Ségonne F., Fischl B., Quinn B. T., Dickerson B. C., Blacker D., Buckner R. L., Dale A. M., Maguire R. P., Hyman B. T., Albert M. S., & Killiany R. J. (2006). An automated labeling system for subdividing the human cerebral cortex on MRI scans into gyral based regions of interest. NeuroImage, 31(3), 968–980. 10.1016/j.neuroimage.2006.01.021 16530430

[R26] Di Vico I. A., Moretto M., Tamanti A., Tomelleri G., Burati G., Martins D., Dipasquale O., Veronese M., Bertoldo A., Menini E., Sandri A., Ottaviani S., Pizzini F. B., Tinazzi M., & Castellaro M. (2025). Molecular-informed network analysis unveils fatigue-related functional connectivity in Parkinson’s disease [I.A. Di Vico, M. Moretto, M. Tinazzi, and M. Castellaro contributed equally to this work]. Movement Disorders. 10.1002/mds.30214 PMC12371637 40260733

[R27] Diedrichsen J. (2009). A spatially unbiased atlas template of the human cerebellum. NeuroImage, 33(1), 127–138. 10.1016/j.neuroimage.2006.05.056 16904911

[R28] Ding Y.-S., Singhal T., Planeta-Wilson B., Gallezot J.-D., Nabulsi N., Labaree D., Wong C., Carson R. E., & Malison R. T. (2010). PET imaging of the effects of age and cocaine on the norepinephrine transporter in the human brain using (S,S)-[11C]O-methylreboxetine and HRRT. Synapse, 64(1), 30–38. 10.1002/syn.20696 19728366 PMC3727644

[R29] Dipasquale O., Cohen A., Martins D., Zelaya F., Turkheimer F., Veronese M., Mehta M. A., Williams S. C. R., Yang B., Banerjee S., & Wang Y. (2023). Molecular-enriched functional connectivity in the human brain using multiband multi-echo simultaneous ASL/BOLD fMRI. Scientific Reports, 13(1), 11751. 10.1038/s41598-023-38573-0 37474568 PMC10359289

[R30] Dipasquale O., Martins D., Sethi A., Veronese M., Hesse S., Rullmann M., Sabri O., Turkheimer F., Harrison N. A., Mehta M. A., & Cercignani M. (2020). Unravelling the effects of methylphenidate on the dopaminergic and noradrenergic functional circuits. Neuropsychopharmacology, 45(9), 1482–1489. 10.1038/s41386-020-0724-x 32473593 PMC7360745

[R31] Dipasquale O., Selvaggi P., Veronese M., Gabay A. S., Turkheimer F., & Mehta M. A. (2019). Receptor-enriched analysis of functional connectivity by targets (REACT): A novel, multimodal analytical approach informed by pet to study the pharmacodynamic response of the brain under MDMA. NeuroImage, 195, 252–260. 10.1016/j.neuroimage.2019.04.007 30953835 PMC6547164

[R32] Dugré M., Chatelain Y., & Glatard T. (2025). An analysis of performance bottlenecks in MRI preprocessing. GigaScience, 14, giae098. 10.1093/gigascience/giae098 PMC11899568 40072903

[R33] Esteban O., Birman D., Schaer M., Koyejo O. O., Poldrack R. A., & Gorgolewski K. J. (2017). MRIQC: Advancing the automatic prediction of image quality in MRI from unseen sites. PLOS ONE, 12(9), e0184661. 10.1371/journal.pone.0184661 28945803 PMC5612458

[R34] Esteban O., Ciric R., Finc K., Blair R. W., Markiewicz C. J., Moodie C. A., Kent J. D., Goncalves M., DuPre E., Gomez D. E. P., Ye Z., Salo T., Valabregue R., Amlien I. K., Liem F., Jacoby N., Stojić H., Cieslak M., Urchs S., … Gorgolewski K. J. (2020). Analysis of taskbased functional MRI data preprocessed with fMRIPrep. Nature Protocols, 15, 2189–2206. 10.1038/s41596-020-0327-3 PMC7404612 32514178

[R35] Esteban O., Markiewicz C. J., Blair R. W., Moodie C. A., Isik A. I., Erramuzpe A., Kent J. D., Goncalves M., DuPre E., Snyder M., Oya H., Ghosh S. S., Wright J., Durnez J., Poldrack R. A., & Gorgolewski K. J. (2019). fMRIPrep: A robust preprocessing pipeline for functional MRI. Nature Methods, 16, 111–116. 10.1038/s41592-018-0235-4 30532080 PMC6319393

[R36] Ferreira L. K., & Busatto G. F. (2013). Resting-state functional connectivity in normal brain aging. Neuroscience & Biobehavioral Reviews, 37(3), 384–400. 10.1016/j.neubiorev.2013.01.017 23333262

[R37] Fischl B. (2012). Freesurfer. NeuroImage, 62, 774–781. 10.1016/j.neuroimage.2012.01.021 22248573 PMC3685476

[R38] Fischl B., Sereno M. I., & Dale A. M. (2002). Whole brain segmentation: Automated labeling of neuroanatomical structures in the human brain. Neuron, 33(3), 341–355. 10.1016/s0896-6273(02)00569-x 11832223

[R39] FMRIB Analysis Group, University of Oxford & Harvard Center for Morphometric Analysis. (2023). Harvard–oxford cortical and subcortical structural atlases. https://fsl.fmrib.ox.ac.uk/fsl/fslwiki/Atlases

[R40] Fonov V., Evans A. C., Botteron K., Almli C. R., McKinstry R. C., & Collins D. L. (2011). Unbiased average age-appropriate atlases for pediatric studies. NeuroImage, 54(1), 313–327. 10.1016/j.neuroimage.2010.07.033 20656036 PMC2962759

[R41] Fortin J.-P., Cullen N., Sheline Y. I., Taylor W. D., Aselcioglu I., Cook P. A., Adams P., Cooper C., Fava M., McGrath P. J., McInnis M., Phillips M. L., Trivedi M. H., Weissman M. M., & Shinohara R. T. (2018). Harmonization of cortical thickness measurements across scanners and sites. NeuroImage, 167, 104–120. 10.1016/j.neuroimage.2017.11.024 29155184 PMC5845848

[R42] Fortin J.-P., Parker D., Tunc B., Watanabe T., Elliott M. A., Ruparel K., Roalf D. D., Satterthwaite T. D., Gur R. C., Gur R. E., Schultz R. T., Verma R., & Shinohara R. T. (2017). Harmonization of multi-site diffusion tensor imaging data. NeuroImage, 161, 149–170. 10.1016/j.neuroimage.2017.08.047 28826946 PMC5736019

[R43] Franke K., & Gaser C. (2012). Longitudinal changes in individual BrainAGE in healthy aging, mild cognitive impairment, and Alzheimer’s disease. GeroPsych, 25(4), 235–245. 10.1024/1662-9647/a000074

[R44] Franke K., & Gaser C. (2019). Ten years of BrainAGE as a neuroimaging biomarker of brain aging: What insights have we gained? Frontiers in Neurology, 10, 789. 10.3389/fneur.2019.00789 31474922 PMC6702897

[R45] Franke K., Gaser C., Manor B., & Novak V. (2013). Advanced BrainAGE in older adults with type 2 diabetes mellitus. Frontiers in Aging Neuroscience, 5, 90. 10.3389/fnagi.2013.00090 24381557 PMC3865444

[R46] Franke K., Ziegler G., Klöppel S., Gaser C., & the Alzheimer’s Disease Neuroimaging Initiative. (2010). Estimating the age of healthy subjects from T1-weighted MRI scans using kernel methods: Exploring the influence of various parameters. NeuroImage, 50(3), 883–892. 10.1016/j.neuroimage.2010.01.005 20070949

[R47] García-Gómez F. J., García-Solís D., Luis-Simón F. J., Marín-Olives J. L., Carrillo F., & Mir P. (2013). Elaboration of an SPM template for the standardization of ^123^I-ioflupane SPECT images. Revista Española de Medicina Nuclear e Imagen Molecular (English Edition), 32(6), 350–356. 10.1016/j.remnie.2013.09.003 23570700

[R48] Gonneaud J., Baria A. T., Pichet Binette A., Gordon B. A., Chhatwal J. P., Cruchaga C., Jucker M., Levin J., Salloway S., Farlow M., Gauthier S., Benzinger T. L. S., Morris J. C., Bateman R. J., Breitner J. C. S., Poirier J., Vachon-Presseau E., Villeneuve S., Alzheimer’s DiseaseNeuroimaging Initiative (ADNI), … Pre-symptomatic Evaluation of Experimental or Novel Treatments for Alzheimer’s Disease (PREVENT-AD) Research Group. (2021). Accelerated functional brain aging in pre-clinical familial alzheimer’s disease. Nature Communications, 12(1), 5346. 10.1038/s41467-021-25492-9 PMC8429427 34504080

[R49] Gorgolewski K. J., Auer T., Calhoun V. D., Craddock R. C., Das S., Duff E. P., Flandin G., Ghosh R. S., Glatard T., Halchenko Y. O., Handwerker D. A., Hanke M., Keator D., Li X., Michael Z., Maumet C., Nichols B. N., Nichols T. E., Pellman J., … Poldrack R. A. (2016). The brain imaging data structure, a format for organizing and describing outputs of neuroimaging experiments. Scientific Data, 3, 160044. 10.1038/sdata.2016.44 27326542 PMC4978148

[R50] Gorgolewski K. J., Burns C. D., Madison C., Clark D., Halchenko Y. O., Waskom M. L., & Ghosh S. S. (2011). Nipype: A flexible, lightweight and extensible neuroimaging data processing framework in python. Frontiers in Neuroinformatics, 5, 13. 10.3389/fninf.2011.00013 21897815 PMC3159964

[R51] Greve D. N., & Fischl B. (2009). Accurate and robust brain image alignment using boundary-based registration. NeuroImage, 48(1), 63–72. 10.1016/j.neuroimage.2009.06.060 19573611 PMC2733527

[R52] Gutiérrez Becker B., Klein T., & Wachinger C. (2018). Gaussian process uncertainty in age estimation as a measure of brain abnormality. NeuroImage, 175, 246–258. 10.1016/j.neuroimage.2018.03.075 29627589

[R53] Han L. K. M., Dinga R., Hahn T., Ching C. R. K., Eyler L. T., Aftanas L., Aghajani M., Aleman A., Baune B. T., Berger K., Brak I., Filho G. B., Carballedo A., Connolly C. G., Couvy-Duchesne B., Cullen K. R., Dannlowski U., Davey C. G., Dima D., … Schmaal L. (2020). Brain aging in major depressive disorder: Results from the ENIGMA major depressive disorder working group. Molecular Psychiatry, 26(9), 5124–5139. 10.1038/s41380-020-0754-0 32424236 PMC8589647

[R54] Hansen J. Y., Shafiei G., Markello R. D., Smart K., Cox S. M. L., Nørgaard M., Beliveau V., Wu Y., Gallezot J.-D., Aumont É., Servaes S., Scala S. G., DuBois J. M., Wainstein G., Bezgin G., Funck T., Schmitz T. W., Spreng R. N., Galovic M., … Misic B. (2022). Mapping neurotransmitter systems to the structural and functional organization of the human neocortex. Nature Neuroscience, 25(11), 1569–1581. 10.1038/s41593-022-01186-3 36303070 PMC9630096

[R55] Harms M. P., Somerville L. H., Ances B. M., Andersson J., Barch D. M., Bastiani M., Bookheimer S. Y., Brown T. B., Buckner R. L., Burgess G. C., Coalson T. S., Chappell M. A., Dapretto M., Douaud G., Fischl B., Glasser M. F., Greve D. N., Hodge C., Jamison K. W., … Yacoub E. (2018). Extending the Human Connectome Project across ages: Imaging protocols for the lifespan development and aging projects. NeuroImage, 183, 972–984. 10.1016/j.neuroimage.2018.09.060 30261308 PMC6484842

[R56] Hesse S., Becker G.-A., Rullmann M., Bresch A., Luthardt J., Hankir M. K., Zientek F., Reißig G., Patt M., Arelin K., Lobsien D., Müller U., Baldofski S., Meyer P. M., Blüher M., Fasshauer M., Fenske W. K., Stumvoll M., Hilbert A., … Sabri O. (2017). Central noradrenaline transporter availability in highly obese, non-depressed individuals. European Journal of Nuclear Medicine and Molecular Imaging, 44(6), 1056–1064. 10.1007/s00259-016-3590-3 28066877 PMC5538358

[R57] Høgestøl E. A., Kaufmann T., Nygaard G. O., Beyer M. K., Sowa P., Nordvik J. E., Kolskår K., Richard G., Andreassen O. A., Harbo H. F., & Westlye L. T. (2019). Cross-sectional and longitudinal MRI brain scans reveal accelerated brain aging in multiple sclerosis. Frontiers in Neurology, 10, 450. 10.3389/fneur.2019.00450 31114541 PMC6503038

[R58] Jenkinson M., Beckmann C. F., Behrens T. E. J., Woolrich M. W., & Smith S. M. (2012). FSL. NeuroImage, 62, 782–790. 10.1016/j.neuroimage.2011.09.015 21979382

[R59] Johnson W. E., Li C., & Rabinovic A. (2007). Adjusting batch effects in microarray expression data using empirical bayes methods. Biostatistics, 8(1), 118–127. 10.1093/biostatistics/kxj037 16632515

[R60] Kanel P., Koeppe R. A., Kotagal V., Roytman S., Muller M. L. T. M., Bohnen N. I., & Albin R. L. (2023). Regional serotonin terminal density in aging human brain: A [^11^C]DASB PET study. Aging Brain, 3, 100071. 10.1016/j.nbas.2023.100071 37408789 PMC10318302

[R61] Karrer T. M., Josef A. K., Mata R., Morris E. D., & Samanez-Larkin G. R. (2017). Reduced dopamine receptors and transporters but not synthesis capacity in normal aging adults: A meta-analysis. Neurobiology of Aging, 57, 36–46. 10.1016/j.neurobiolaging.2017.05.006 28599217 PMC5645072

[R62] Klein A., & Tourville J. (2012). 101 labeled brain images and a consistent human cortical labeling protocol. Frontiers in Neuroscience, 6, 171. 10.3389/fnins.2012.00171 23227001 PMC3514540

[R63] Koutsouleris N., Davatzikos C., Borgwardt S., Gaser C., Bottlender R., Frodl T., Falkai P., Riecher-Rossler A., Moller H.-J., Reiser M., Pantelis C., & Meisenzahl E. (2013). Accelerated brain aging in schizophrenia and beyond: A neuroanatomical marker of psychiatric disorders. Schizophrenia Bulletin, 40(5), 1140–1153. 10.1093/schbul/sbt142 24126515 PMC4133663

[R64] Lawn T., Dipasquale O., Vamvakas A., Tsougos I., Mehta M. A., & Howard M. A. (2022). Differential contributions of serotonergic and dopaminergic functional connectivity to the phenomenology of LSD. Psychopharmacology, 239(6), 1797–1808. 10.1007/s00213-022-06117-5 35322297 PMC9166846

[R65] Lawn T., Giacomel A., Martins D., Veronese M., Howard M., Turkheimer F. E., & Dipasquale O. (2024). Normative modelling of molecular-based functional circuits captures clinical heterogeneity transdiagnostically in psychiatric patients. Communications Biology, 7(689). 10.1038/s42003-024-06391-3 PMC11153627 38839931

[R66] Liem F., Varoquaux G., Kynast J., Beyer F., Kharabian Masouleh S., Huntenburg J. M., Lampe L., Rahim M., Abraham A., Craddock R. C., Riedel-Heller S., Luck T., Loeffler M., Schroeter M. L., Witte A. V., Villringer A., & Margulies D. S. (2017). Predicting brain-age from multimodal imaging data captures cognitive impairment. NeuroImage, 148, 179–188. 10.1016/j.neuroimage.2016.11.005 27890805

[R67] Livingston G., Huntley J., Liu K. Y., Costafreda S. G., Selbæk G., Alladi S., Ames D., Banerjee S., Burns A., Brayne C., Fox N. C., Ferri C. P., Gitlin L. N., Howard R., Kales H. C., Kivimäki M., Larson E. B., Nakasujja N., Rockwood K., … Mukadam N. (2024). Dementia prevention, intervention, and care: 2024 report of the Lancet standing commission. The Lancet, 404(10452), 572–628. 10.1016/s0140-6736(24)01296-0 39096926

[R68] Logothetis N. K. (2008). What we can do and what we cannot do with fMRI. Nature, 453(7197), 869–878. 10.1038/nature06976 18548064

[R69] MacDonald M. E., & Pike G. B. (2021). MRI of healthy brain aging: A review. NMR in Biomedicine, 34(9), e4564. 10.1002/nbm.4564 34096114

[R70] Martins D., Veronese M., Turkheimer F. E., Howard M. A., Williams S. C. R., & Dipasquale O. (2021). A candidate neuroimaging biomarker for detection of neurotransmission-related functional alterations and prediction of pharmacological analgesic response in chronic pain. Brain Communications, 4(1). 10.1093/braincomms/fcab302 PMC8833258 35169702

[R71] Mather M., & Harley C. W. (2016). The locus coeruleus: Essential for maintaining cognitive function and the aging brain. Trends in Cognitive Sciences, 20(3), 214–226. 10.1016/j.tics.2016.01.001 26895736 PMC4761411

[R72] Mehta K., Salo T., Madison T. J., Adebimpe A., Bassett D. S., Bertolero M., Cieslak M., Covitz S., Houghton A., Keller A. S., Lundquist J. T., Luo A., Miranda-Dominguez O., Nelson S. M., Shafiei G., Shanmugan S., Shinohara R. T., Smyser C. D., Sydnor V. J., … Satterthwaite T. D. (2024). XCP-D: A robust pipeline for the post-processing of fMRI data. Imaging Neuroscience, 2. 10.1162/imag_a_00257 PMC12288603 40800264

[R73] Millar P. R., Gordon B. A., Luckett P. H., Benzinger T. L., Cruchaga C., Fagan A. M., Hassenstab J. J., Perrin R. J., Schindler S. E., Allegri R. F., Day G. S., Farlow M. R., Mori H., Nübling G., Bateman R. J., Morris J. C., & Ances B. M. (2023). Multimodal brain age estimates relate to Alzheimer disease biomarkers and cognition in early stages: A cross-sectional observational study. eLife, 12. 10.7554/elife.81869 PMC9988262 36607335

[R74] Millar P. R., Luckett P. H., Gordon B. A., Benzinger T. L. S., Schindler S. E., Fagan A. M., Cruchaga C., Bateman R. J., Allegri R. F., Jucker M., Lee J.-H., Mori H., Salloway S. P., Yakushev I., Morris J. C., Ances B. M., & Dominantly Inherited Alzheimer Network. (2022). Predicting brain age from functional connectivity in symptomatic and preclinical Alzheimer disease [Epub 2022 Apr 16]. NeuroImage, 256, 119228. 10.1016/j.neuroimage.2022.119228 35452806 PMC9178744

[R75] Moretto M., Silvestri E., Zangrossi A., Corbetta M., & Bertoldo A. (2022). Unveiling whole-brain dynamics in normal aging through hidden Markov models. Human Brain Mapping, 43(3), 1129–1144. 10.1002/hbm.25714 34783122 PMC8764474

[R76] Moskalev A. (Ed.). (2019). Biomarkers of human aging. Springer International Publishing. 10.1007/978-3-030-24970-0

[R77] Nakua H., Hawco C., Forde N. J., Joseph M., Grillet M., Johnson D., Jacobs G. R., Hill S., Voineskos A. N., Wheeler A. L., Lai M.-C., Szatmari P., Georgiades S., Nicolson R., Schachar R., Crosbie J., Anagnostou E., Lerch J. P., Arnold P. D., & Ameis S. H. (2023). Systematic comparisons of different quality control approaches applied to three large pediatric neuroimaging datasets. NeuroImage, 274, 120119. 10.1016/j.neuroimage.2023.120119 37068719

[R78] Nenadić I., Dietzek M., Langbein K., Sauer H., & Gaser C. (2017). BrainAGE score indicates accelerated brain aging in schizophrenia, but not bipolar disorder. Psychiatry Research: Neuroimaging, 266, 86–89. 10.1016/j.pscychresns.2017.05.006 28628780

[R79] Nitsch A., Garvert M. M., Bellmund J. L. S., Schuck N. W., & Doeller C. F. (2024). Grid-like entorhinal representation of an abstract value space during prospective decision making. Nature Communications, 15, 1198. 10.1038/s41467-024-45127-z PMC10858181 38336756

[R80] Nooner K. B., Colcombe S. J., Tobe R. H., Mennes M., Benedict M. M., Moreno A. L., Panek L. J., Brown S., Zavitz S. T., Li Q., Sikka S., Gutman D., Bangaru S., Schlachter R. T., Kamiel S. M., Anwar A. R., Hinz C. M., Kaplan M. S., Rachlin A. B., … Milham M. P. (2012). The NKI–Rockland sample: A model for accelerating the pace of discovery science in psychiatry. Frontiers in Neuroscience, 6, 152. 10.3389/fnins.2012.00152 23087608 PMC3472598

[R81] Pardoe H. R., Cole J. H., Blackmon K., Thesen T., Kuzniecky R., & Human Epilepsy Project Investigators. (2017). Structural brain changes in medically refractory focal epilepsy resemble premature brain aging. Epilepsy Research, 133, 28–32. 10.1016/j.eplepsyres.2017.03.007 28410487

[R82] Pinamonti M., Sammassimo V., Moretto M., & Veronese M. (2025). Comparison of statistical methods for brain age prediction using neuroimaging data [Accepted for publication; M. Pinamonti and V. Sammassimo contributed equally to this work]. Proceedings of the 47th IEEE Engineering in Medicine and Biology Conference, to appear. 10.1109/EMBC58623.2025.11254883 41335739

[R83] Power J. D., Barnes K. A., Snyder A. Z., Schlaggar B. L., & Petersen S. E. (2012). Spurious but systematic correlations in functional connectivity MRI networks arise from subject motion. NeuroImage, 59(3), 2142–2154. 10.1016/j.neuroimage.2011.10.018 22019881 PMC3254728

[R84] Provins C., MacNicol E., Seeley S. H., Hagmann P., & Esteban O. (2023). Quality control in functional MRI studies with MRIQC and fMRIPrep. Frontiers in Neuroimaging, 1, 1073734. 10.3389/fnimg.2022.1073734 37555175 PMC10406249

[R85] Puledda F., Dipasquale O., Gooddy B. J. M., Karsan N., Bose R., Mehta M. A., Williams S. C. R., & Goadsby P. J. (2023). Abnormal glutamatergic and serotonergic connectivity in visual snow syndrome and migraine with aura. Annals of Neurology, 94(5), 873–884. 10.1002/ana.26745 37466404

[R86] Satterthwaite T. D., Elliott M. A., Gerraty R. T., Ruparel K., Loughead J., Calkins M. E., Eickhoff S. B., Hakonarson H., Gur R. C., Gur R. E., & Wolf D. H. (2013). An improved framework for confound regression and filtering for control of motion artifact in the preprocessing of resting-state functional connectivity data. NeuroImage, 64, 240–256. 10.1016/j.neuroimage.2012.08.052 22926292 PMC3811142

[R87] Schaefer A., Kong R., Gordon E. M., Laumann T. O., Zuo X.-N., Holmes A. J., Eickhoff S. B., & Yeo B. T. T. (2017). Local-global parcellation of the human cerebral cortex from intrinsic functional connectivity mri. Cerebral Cortex, 28(9), 3095–3114. 10.1093/cercor/bhx179 PMC6095216 28981612

[R88] Schnack H. G., van Haren N. E. M., Nieuwenhuis M., Hulshoff Pol H. E., Cahn W., & Kahn R. S. (2016). Accelerated brain aging in schizophrenia: A longitudinal pattern recognition study. American Journal of Psychiatry, 173(6), 607–616. 10.1176/appi.ajp.2015.15070922 26917166

[R89] Shafto M. A., Tyler L. K., Dixon M., Taylor J. R., Rowe J. B., Cusack R., Calder A. J., Marslen-Wilson W. D., Duncan J., Dalgleish T., Henson R. N., Brayne C., Matthews F. E., & Cam-CAN. (2014). The Cambridge centre for ageing and neuroscience (Cam-CAN) study protocol: A cross-sectional, lifespan, multidisciplinary examination of healthy cognitive ageing. BMC Neurology, 14(1), 204. 10.1186/s12883-014-0204-1 25412575 PMC4219118

[R90] Singleton S. P., Wang J. B., Mithoefer M., Hanlon C., George M. S., Mithoefer A., Mithoefer O., Coker A. R., Yazar-Klosinski B., Emerson A., Doblin R., & Kuceyeski A. (2023). Altered brain activity and functional connectivity after MDMA-assisted therapy for post-traumatic stress disorder. Frontiers in Psychiatry, 13. 10.3389/fpsyt.2022.947622 PMC9879604 36713926

[R91] Smith S. M., Jenkinson M., Woolrich M. W., Beckmann C. F., Behrens T. E., Johansen-Berg H., Bannister P. R., De Luca M., Drobnjak I., Flitney D. E., Niazy R. K., Saunders J., Vickers J., Zhang Y., De Stefano N., Brady J. M., & Matthews P. M. (2004). Advances in functional and structural MR image analysis and implementation as FSL. NeuroImage, 23, S208–S219. 10.1016/j.neuroimage.2004.07.051 15501092

[R92] Taylor J. R., Williams N., Cusack R., Auer T., Shafto M. A., Dixon M., Tyler L. K., Cam-CAN, & Henson R. N. (2017). The Cambridge centre for ageing and neuroscience (Cam-CAN) data repository: Structural and functional MRI, MEG, and cognitive data from a cross-sectional adult lifespan sample. NeuroImage, 144, 262–269. 10.1016/j.neuroimage.2015.09.018 26375206 PMC5182075

[R93] Tian L., Li Q., Wang C., & Yu J. (2018). Changes in dynamic functional connections with aging. NeuroImage, 172, 31–39. 10.1016/j.neuroimage.2018.01.040 29414496

[R94] Tobe R. H., MacKay-Brandt A., Lim R., Kramer M., Breland M. M., Tu L., Tian Y., Trautman K. D., Hu C., Sangoi R., Alexander L., Gabbay V., Castellanos F. X., Leventhal B. L., Craddock R. C., Colcombe S. J., Franco A. R., & Milham M. P. (2022). A longitudinal resource for studying connectome development and its psychiatric associations during childhood. Scientific Data, 9(1). 10.1038/s41597-022-01329-y PMC9197863 35701428

[R95] Tukey J. W. (1977). Exploratory data analysis. Addison–Wesley.

[R96] Tustison N. J., Avants B. B., Cook P. A., Zheng Y., Egan A., Yushkevich P. A., & Gee J. C. (2010). N4ITK: Improved N3 bias correction. IEEE Transactions on Medical Imaging, 29(6), 1310–1320. 10.1109/tmi.2010.2046908 20378467 PMC3071855

[R97] Udeh-Momoh C. T., Migeot J., Blackmon K., Mielke M. M., Melloni M., Cox L., Yaffe K., Santamaria-Garcia H., Stern Y., & Ibanez A. (2025). Resilience and brain health in global populations. Nature Medicine, 31(8), 2518–2531. 10.1038/s41591-025-03846-w PMC13189249 40731089

[R98] United Nations, Department of Economic and Social Affairs, Population Division. (2019). World population ageing 2019: Highlights. https://www.un.org/en/development/desa/population/publications/pdf/ageing/WorldPopulationAgeing2019-Highlights.pdf

[R99] Varoquaux G. (2018). Cross-validation failure: Small sample sizes lead to large error bars. NeuroImage, 180, 68–77. 10.1016/j.neuroimage.2017.06.061 28655633

[R100] Wang J., Knol M. J., Tiulpin A., Dubost F., de Bruijne M., Vernooij M. W., Adams H. H. H., Ikram M. A., Niessen W. J., & Roshchupkin G. V. (2019). Gray matter age prediction as a biomarker for risk of dementia. Proceedings of the National Academy of Sciences of the United States of America, 116(42), 21213–21218. 10.1073/pnas.1902376116 31575746 PMC6800321

[R101] World Health Organization. (2024). Ageing and health. https://www.who.int/news-room/fact-sheets/detail/ageing-and-health

[R102] Xia Y., Chen Q., Shi L., Li M., Gong W., Chen H., & Qiu J. (2019). Tracking the dynamic functional connectivity structure of the human brain across the adult lifespan. Human Brain Mapping, 40(3), 717–728. 10.1002/hbm.24385 30515914 PMC6865727

[R103] Yu Y., Cui H.-Q., Haas S. S., New F., Sanford N., Yu K., Zhan D., Yang G., Gao J.-H., Wei D., Qiu J., Banaj N., Boomsma D. I., Breier A., Brodaty H., Buckner R. L., Buitelaar J. K., Cannon D. M., Caseras X., … ENIGMA-Lifespan Working Group. (2024). Brain-age prediction: Systematic evaluation of site effects, and sample age range and size. Human Brain Mapping, 45(10), e26768. 10.1002/hbm.26768 38949537 PMC11215839

[R104] Zhai J., & Li K. (2019). Predicting brain age based on spatial and temporal features of human brain functional networks. Frontiers in Human Neuroscience, 13, 62. 10.3389/fnhum.2019.00062 30863296 PMC6399206

[R105] Zhang Y., Brady M., & Smith S. (2001). Segmentation of brain MR images through a hidden Markov random field model and the expectation-maximization algorithm. IEEE Transactions on Medical Imaging, 20(1), 45–57. 10.1109/42.906424 11293691

