# Supplementary material for: Investigating the Contribution of Molecular-Enriched Functional Connectivity to Brain-Age Analysis
Source: bioRxiv. 2025 Oct 16:2025.10.16.682939. Preprint. [Version 1] doi: 10.1101/2025.10.16.682939 (PMC12632960; doi:10.1101/2025.10.16.682939)
Supplement: 1 [file NIHPP2025.10.16.682939V1-supplement-1.pdf]

bioRxiv preprint doi: https://doi.org/10.1101/2025.10.16.682939; this version posted October 16, 2025. The copyright holder for this preprint (which was not certified by peer review) is the author/funder, who has granted bioRxiv a license to display the preprint in perpetuity. It is made available under aCC-BY 4.0 International license.

Supplementary Material

Subjects excluded

Quality control of raw images

Automated MRIQC screening flagged a subset of scans with poor signal-to-noise ratio, motion artifacts, or other anomalies; those participants were removed before preprocessing. In total, 61 individuals were excluded: 51 because of structural issues and 10 because of functional issues. The dataset-specific counts are listed in Supplementary Table S1.

Table S1: Participants removed after MRIQC

Dataset	Structural (T1w)	Functional (rs-fMRI)	Total
Cam-CAN	3	2	5
HCP-Aging	25	8	33
NKI-RS	23	0	23
**Total**	**51**	**10**	**61**

Preprocessing outputs

During structural and functional MRI preprocessing, a small additional fraction of scans failed because of unrecoverable software errors or excessive motion. Specifically, *fMRIPrep* terminated early for five HCP-Aging participants, whereas no such failures occurred in Cam-CAN or NKI-RS. Subsequent denoising with XCP-D led to the exclusion of participants whose residual time series contained more than one-third of volumes with framewise displacement exceeding 0.5 mm. Finally, one subject was excluded from Cam-CAN sample because `react-fmri` did not run successfully on it. Table S2 details these counts: in total, 165 participants were removed at the preprocessing stage, leaving 2128 individuals for downstream analyses. This number lowers to 2120 after the exclusion of HCP-Aging participants over 90 years old: this final sample constitutes the dataset used for this study.

Molecular-enriched functional connectivity

Only one run failed (subject from Cam-CAN) and thus that participant was excluded from the analysis.

bioRxiv preprint doi: https://doi.org/10.1101/2025.10.16.682939; this version posted October 16, 2025. The copyright holder for this preprint (which was not certified by peer review) is the author/funder, who has granted bioRxiv a license to display the preprint in perpetuity. It is made available under aCC-BY 4.0 International license.

Table S2: Participants excluded during preprocessing.

**Dataset**	**fMRIPrep**	**XCP-D**	**REACT**	**Total**
Cam-CAN	0	20	1	21
HCP-Aging	5	79	0	84
NKI-RS	0	60	0	60
Total	5	159	1	165
